# Application of High-Flow Nasal Cannula in COVID-19: A Narrative Review

**DOI:** 10.3390/life12091419

**Published:** 2022-09-12

**Authors:** Cheng-Wei Liu, Shih-Lung Cheng

**Affiliations:** 1 Department of Internal Medicine, Far Eastern Memorial Hospital, New Taipei City 22000, Taiwan; 2 Graduate Institute of Medicine, Yuan-Ze University, Taoyuan City 32003, Taiwan

**Keywords:** high-flow nasal cannula, COVID-19, SARS-CoV-2, acute hypoxemic respiratory failure

## Abstract

Background: During the first wave of COVID-19, the large influx of severely ill patients led to insufficient availability of beds in intensive care units and a shortage of ventilators. The shortage of ventilators, high mortality of intubated patients, and high risk of infections among healthcare workers involved in intubation were the main factors that led to the prevalence of noninvasive respiratory support during the pandemic. The high-flow nasal cannula (HFNC) is a commonly used, popular form of noninvasive respiratory support. Due to its unique physiological effects, HFNC can provide a high fraction of humidified oxygen and is satisfactorily comfortable for patients with COVID-19. However, before the COVID-19 era, there was little evidence on the application of HFNC in patients with acute respiratory failure caused by viral infection. Aim: This narrative review provides an overview of recent studies on the use of HFNC in patients with COVID-19-related acute hypoxemic respiratory failure. The main topics discussed include the probability of successful use of HFNC in these patients, whether late intubation increases mortality, the availability of convenient and accurate monitoring tools, comparison of HFNC with other types of noninvasive respiratory support, whether HFNC combined with the prone position is more clinically useful, and strategies to further reduce the infection risk associated with HFNC. The implication of this study is to identify some of the limitations and research gaps of the current literature and to give some advice for future research.

## 1. Introduction

The first known case of coronavirus disease (COVID-19) caused by the novel coronavirus (SARS-CoV-2) was reported at the end of 2019 in Wuhan, Hubei Province, China. After the first outbreak in Wuhan, the number of cases increased rapidly throughout the world. To date, there have been more than 570 million confirmed cases of COVID-19 and 6.4 million deaths according to the WHO Coronavirus (COVID-19) Dashboard [1].

Patients with COVID-19 have a wide range of symptoms, including fever or chills, coughing, shortness of breath, muscle aches, sore throat, and diarrhea. SARS-CoV-2 may also invade the CNS (central nervous system) through the nasal cavity and cause CNS symptoms including new loss of smell and taste, headache, fatigue, and unconsciousness [2]. Severe cases may develop acute hypoxemic respiratory failure (AHRF), which requires O_2_ support. COVID-19 ARDS (acute respiratory distress syndrome) is not a typical ARDS because of its normal lung compliance in the early stage (i.e., phynotype 1) and anti-gravitational regional blood flow [3]. Intubation with mechanical ventilation may be considered for the later stage of COVID-19 ARDS (i.e., phynotype 2), to decrease the work of breathing and provide adequate oxygen [3].

An early retrospective, observational study of 52 hypoxic patients at an intensive care unit (ICU) reported an invasive mechanical ventilation (IMV) rate of 42% [4]. Although the IMV rate may have varied among different countries or hospitals due to the availability of ventilators, COVID-19-related AHRF imposed a severe strain on ICU resources worldwide and many hospitals faced a shortage of beds and ventilators [5]. Moreover, high mortality rates were reported for patients who underwent IMV [6,7,8]. An observational study by Blonz et al. observed a high incidence of ventilator-associated pneumonia (VAP) in patients with COVID-19 who received IMV [9]; VAP is associated with increased mortality among patients who undergo IMV [10].

Due to these factors, noninvasive respiratory support may be considered for COVID-19-related AHRF. The high-flow nasal cannula (HFNC) is a popular modality used for patients with hypoxemic respiratory failure as it is noninvasive, easy to set, and can be used outside the ICU setting. Several studies have evaluated the effectiveness of HFNC in patients with COVID-19-related AHRF. The aim of this review is to summarize the available evidence on the use of HFNC in patients with COVID-19-related AHRF and to point to some limitations and gaps in the literature and to give some suggestions for future research.

## 2. Methods

We searched PubMed and MEDLINE using the search terms: (“high-flow nasal cannula” or “HFNC” or “HFNO”) and (“COVID-19” or “Coronavirus” or “SARS-CoV-2”). We focused on observational studies, clinical trials, randomized control trials (RCTs), guidelines, and meta-analyses; other similar types of articles were also eligible for this review.

## 3. HFNC: Past Evidence and Experience

HFNC is used to deliver warm, humidified oxygen at high flow rates (20–60 L/min) and can enable alveolar FiO_2_ to achieve a defined value. The physiological effects of HFNC include increased oropharyngeal airway pressure, end-expiratory lung volume (EELV), and tidal volume, which collectively decrease the respiratory rate, reduce dyspnea, and improve oxygenation. In addition, HFNC is able to provide low positive end expiratory pressure (PEEP), which can reduce airway pressure, increase oxygenation, and decrease the work of breathing. In contrast to noninvasive ventilation (NIV), HFNC is an open system that allows the patient to speak, cough, and eat. HFNC has many physiological benefits and is comfortable; thus, it has been increasingly used in patients with AHRF in recent years.

A number of studies have explored the effectiveness and comfort of HFNC compared to conventional oxygen therapy or NIV. Roca et al. [11] demonstrated that HFNC was more comfortable and led to better oxygenation and a reduced respiratory rate compared to conventional oxygen masks. Similar results were reported by Frat et al. [12], who found that HFNC was better than standard oxygen or NIV in terms of improved respiratory discomfort. A randomized non-inferiority trial assessing reintubation and post-extubation respiratory failure in high-risk patients who had undergone extubation reported that HFNC was not inferior to NIV [13]. Another multicenter RCT that evaluated patients presenting to the emergency department with AHRF of any cause also found that HFNC was non-inferior to NIV in terms of avoidance of intubation [14]. A systematic review demonstrated that, compared to conventional oxygen therapy, HFNC may decrease the intubation rate in patients with AHRF of any cause, although the mortality rate was similar between the two treatment groups [15].

In summary, HFNC appears to be a good option for patients with AHRF, as it appears to be as effective as NIV and is more comfortable.

## 4. Current Evidence on the Use of HFNC in COVID-19

### 4.1. Experience of HFNC in Patients with COVID-19

There is a growing body of literature exploring the effectiveness of HFNC in patients with COVID-19-related AHRF. Most of these studies defined intubation, death, or escalation of respiratory support as failure of HFNC. The success rate of HFNC varies between studies.

A prospective multicenter observational study performed in South Africa reported that HFNC had a success rate of 47% (137/292) in patients with COVID-19-related AHRF [16]. A similar success rate (47.5%) was also reported by another retrospective study [17]. A small retrospective observational study by Wang et al. [18] found that HFNC had a success rate of 59% (10/17) in patients with COVID-19-related AHRF. Interestingly, in this study, all patients in whom HFNC failed were escalated to NIV for rescue, instead of intubation. The authors also found that HFNC was used more frequently than NIV as initial oxygen support for COVID-19-related AHRF, probably as HFNC is more comfortable than NIV and is easier to set up.

### 4.2. Wait-and-See Strategy with HFNC vs. Intubation at the Beginning

According to the “patient–self-inflicted lung injury” (P-SILI) theory, an excessive increase in transpulmonary pressure during labored spontaneous breathing may worsen lung damage and increase mortality [19]. Thus, clinicians may ask whether or not intubation at the beginning of AHRF is better than trying HFNC first. Could HFNC be trialed at the beginning of COVID-19-related AHRF and then wait until HFNC fails? This so-called wait-and-see strategy is especially important in resource-constrained settings.

Ricard et al. [20] aimed to address this issue in a prospective multicenter cohort study of 122 patients with COVID-19-related AHRF admitted to ICU, 61 of whom received HFNC on day 1, and the remainder were intubated on day 1. Propensity score matched analysis was used to eliminate possible confounding factors. Compared to the group intubated on day one, the HFNC group had a higher number of ventilator-free days (mean difference: 8 days; 95% CI: 4.4–11.7 days), reduced length of ICU stays (−8.2 days; 95% CI: −12.7 to −3.6 days), and HFNC was not associated with increased hospital all-cause mortality (OR: 0.64; 95% CI: 0.25–4.64). Similar results were also obtained in sensitivity analysis.

Thus, the results of this study may justify the wait-and-see strategy. However, it must be noted that the patients who were intubated on day 1 may have been more seriously ill and this difference may not have been eliminated by statistical adjustment.

### 4.3. The Early vs. Late Intubation Mortality Debate

In the pre-COVID-19 era, a secondary data analysis of a prospective cohort study of patients with ARDS reported that late intubation was associated with increased mortality [21]. Thus, late-intubation-related increased mortality is also a concern for patients with COVID-19-related AHRF.

Hyman et al. [22] retrospectively analyzed 755 intubated patients with COVID-19 and found that intubation increased the in-hospital mortality rate by 1.03-fold per day of delay. However, only 11.7% (88/755) of the patients in that study received HFNC before intubation. A meta-analysis of twelve non-randomized cohort studies that included 8944 critically ill patients with COVID-19 classified intubated patients into the early intubation (within 24 h of admission to ICU) and late intubation groups [23]. No significant differences in all-cause mortality (3981 deaths; 45.4% vs. 39.1%; RR: 1.07, 95% CI: 0.99–1.15, *p* = 0.08) or the duration of mechanical ventilation (1892 patients; MD: −0.58 days, 95% CI: 3.06 to 1.89 days, *p* = 0.65) were observed between the early and late intubation groups. The results of this meta-analysis favored the wait-and-see strategy, in contrast to the study conducted by Hyman et al. [22]. However, not every patient in these studies had a prior trial of HFNC before intubation. Prior trials of different types of noninvasive respiratory support are likely to have confounded the analyses. Thus, the most interesting question is whether prior trial of HFNC affects the differences in the mortality rate between the early intubation and late intubation groups.

Baek et al. [24] performed a multicenter retrospective cohort study to investigate the differences in mortality between early and late intubation in patients with COVID-19-related AHRF who were initially treated with HFNC. Of the 133 patients, HFNC was successful in 63 (47.3%) and failed in 70 (52.6%). Patients in whom HFNC failed were classified into the early failure group (intubation within 48 h of starting HFNC; 50 (71.4%) patients) and late failure group (20; 28.6%). The overall in-hospital mortality rate was 45.7% (32/70). Mortality was higher in the late failure group than early failure group (65.0% vs. 38.0%, *p* = 0.041). Interestingly, the early failure group had a higher Sequential Organ Failure Assessment (SOFA) score than the late failure group, indicating that the patients in the early failure group were more severely ill. The late failure group initially had a lower SOFA score than the early failure group but had a higher late mortality rate than the early failure group. The authors concluded that the late failure group may have had a higher mortality rate.

Another retrospective observational study of 272 patients with COVID-19-related AHRF who received HFNC also used the 48 h boundary to subdivide the failure group into early failure and late failure groups [25]. The overall mortality rate of the failure group was 45.4%, which is similar to the rate reported by Baek et al. However, in contrast to the study conducted by Baek et al., the difference in the in-hospital mortality rate between the early failure group and late failure group was not statistically significant (39.3% vs. 53.2%, *p* = 0.18) (Table 1).

However, both retrospective studies had inconsistent conclusions regarding whether late intubation increases the mortality risk compared to early intubation in patients with COVID-19 who were already treated with HFNC. There are also several limitations to these studies. First, missing data and confounding factors may exist in retrospective studies, and confounding factors are not easily statistically eliminated. Second, the indications for HFNC and protocols for intubation may also vary between centers. Although there is no clear answer as to whether delayed intubation increases the mortality rate in patients with COVID-19 who have already received HFNC, it is important to monitor these patients closely. Thus, well-designed prospective trials of patients with COVID-19-related AHRF who receive a trial of HFNC are needed to address this issue.

### 4.4. Value of the ROX Index for Monitoring Patients Treated with HFNC

When HFNC is applied to patients with AHRF, clinicians may want to predict whether treatment will succeed or fail. Roca et al. [26] performed a prospective cohort study of patients with severe pneumonia treated with HFNC. This was the first study to assess the value of the ROX index to predict which patients could continue to use HFNC. This study showed that an ROX index greater than or equal to 4.88 after 12 h of HFNC (ROX_12h_) was associated with a lower risk of intubation. The ROX index is defined as the ratio of SpO_2_/FiO_2_ to the respiratory rate. All three components of the ROX index can be easily and conveniently measured bedside, and the ROX index can also be used in non-ICU settings.

Hu et al. [27] conducted a retrospective observational study in non-ICU settings that included 105 patients with COVID-19-related AHRF treated with HFNC, in whom HFNC was successful in 65 (62%) patients and failed in 40 (38%) patients. After application of HFNC for two hours, there were no differences in respiratory variables (RR, SpO_2_/FiO_2_, PaO_2_/FiO_2_, or the ROX index) between the success and failure groups. However, after six hours of HFNC, all respiratory variables had increased in the success group and decreased in the failure group. All of the respiratory variables (SpO_2_/FiO_2_, PaO_2_/FiO_2_, and ROX index) at 6 h after initiation of HFNC had good predictive accuracy for the success of HFNC (AUROC: 0.786, 0.749, and 0.798, respectively). Moreover, younger age, female sex, lower SOFA, and a higher ROX index were independent predictors for the success of HFNC. Among these indicators, an ROX_6h_ over 5.55 had the highest odds ratio (OR: 17.821; 95% CI: 3.741–84.903, *p* < 0.001) for the success of HFNC. The authors concluded that the application of HFNC required close monitoring and that an ROX_6h_ cut-off of 5.55 was a good indicator of treatment success.

Another retrospective observational study carried out by Vega et al. [28] included 120 patients with COVID-19-related AHRF treated with HFNC outside the ICU. Although this was a retrospective study, the data were collected prospectively. Of the 120 patients, treatment failed in 35 (29%) patients, who were escalated to IMV. The median time-to-intubation was two days (IQR: 1–3). ROC analysis showed that an ROX_12h_ cut-off value of 5.99 was the best predictor of intubation (AUC: 0.7916, 95% CI: 0.6905–0.8927; specificity: 96%, sensitivity: 62%). Kaplan–Meier curves revealed that an ROX_12h_ below 5.99 was associated with an increased risk of failure of HFNC treatment (*p* = 0.008, log-rank test). Vega et al. concluded that the ROX index may help clinicians to predict patients for whom HFNC would fail and for whom intubation may be indicated, especially in the non-ICU setting of this study.

In addition to the ROX_6h_ or ROX_12h_ cut-off values, Ferrer et al. [29] identified that an ROX_24h_ above 5.35 could predict the success of HFNC, and Panadero et al. [17] reported that an ROX_2–6h_ below 4.94 was associated with an increased risk of intubation (HR: 4.03, 95% CI: 1.18–13.7, *p* = 0.026). The good predictive value of the ROX index for failure of HFNC in patients with COVID-19-related AHRF was further supported by a systematic review reported by Prakash et al. [30].

However, future well-designed prospective trials are required to identify a uniform ROX index cut-off value at a specific time point to better predict which patients in whom HFNC may fail. However, this raises the question of the protocol for patients who have an ROX index below the cut-off value at the given time point. Should these patients be intubated immediately, or should a wait-and-see strategy be adopted until the patients meet the criteria for intubation? The available literature has not addressed this question; thus, future RCTs are also required to answer this issue.

### 4.5. Comparison of Various Types of Noninvasive Respiratory Support

Clinicians may ask whether HFNC is better than other types of noninvasive respiratory support in patients with COVID-19-related AHRF. Demoule et al. [31] performed a retrospective observational study of 379 patients with severe COVID-19 in an ICU setting. The aim of this study was to compare HFNC with conventional oxygen therapy (COT) in terms of the risk of intubation and mortality. After adjustment by propensity score matching, the intubation rate at day 28 was lower in the HFNC group than the COT group (55% (95% CI: 46–63) vs. 72% (95% CI: 64–79), *p* < 0.0001); however, mortality at day 28 was not significantly different (HFNC: 21% vs. COT: 22%; HR: 1.35, 95% CI: 0.56–3.26). The results of this study are in agreement with a previous systematic review [15].

Another retrospective observational study by Hacquin [32] that included older patients (>75 y/o) with COVID-19-related AHRF in non-ICU settings also aimed to compare HFNC with COT. This study found that HFNC may reduce mortality at day 30 compared to COT (weighted HR: 0.57, 95% CI: 0.33–0.99, *p* = 0.04). This study also found that HFNC was associated with less discomfort and lower morphine requirements compared with COT (weighted HR: 0.39, 95% CI: 0.21–0.71, *p* = 0.002). As all subjects in this study were above 75 years old and were cared for in a non-ICU setting, the authors emphasized that their findings indicated that HFNC can be safely used in older patients with COVID-19 in non-ICU settings.

An RCT reported by Teng et al. [33] included 22 patients with severe COVID-19, twelve of whom received HFNC and 10 of whom received COT. After 6 h of treatment, parameters including heart rate (HR), respiratory rate (RR), and PaO_2_/FiO_2_ were better in the HFNC group than the COT group. PaO_2_/FiO_2_ remained significantly higher in the HFNC group than the COT group at both 24 h and 72 h.

Moreover, the HFNC group had a shorter ICU stay and total hospital stay than the COT group. Another RCT of 200 patients with severe COVID-19 compared HFNC with COT in terms of intubation and clinical recovery [34]. Compared to COT, HFNC was associated with a lower intubation rate within 28 days (34.3% vs. 51%; HR: 0.62, 95% CI: 0.39–0.96, *p* = 0.03) and shorter median time to clinical recovery within 28 days (11 days (IQR: 9–14) vs. 14 days (IQR: 11–19); HR: 1.39; 95% CI: 1.00–1.92, *p* = 0.047).

A multicenter retrospective cohort study of 1093 ICU patients with COVID-19-related AHRF analyzed the intubation and mortality rates for patients receiving different types of noninvasive respiratory support [35]. Lower intubation rates were noted in the HFNC group and NIV group than the COT group (70% vs. 88% vs. 91%, respectively; *p* < 0.001). Moreover, HFNC was associated with lower ICU mortality than COT (HR: 0.75 (95% CI: 0.58–0.98)), though NIV was not associated with a lower mortality rate than COT (HR: 1.21 (95% CI: 0.8–1.83)).

However, the recent RECOVERY-RS RCT [36] observed no differences in the intubation or mortality rates within 30 days between the HFNC and COT groups (composite outcome, HFNC vs. COT: 44.3% vs. 45.1%; absolute difference, −1% (95% CI: −8% to 6%), *p* = 0.83). Instead, CPAP led to a better outcome in terms of tracheal intubation or mortality within 30 days than COT (36.3% vs. 44.4%, absolute difference, −8% (95% CI: −15% to −1%), *p* = 0.03). This result of the RECOVERY-RS trial in terms of avoidance of intubation for HFNC is not in line with most previous studies. This difference may be explained by the fact that the study was underpowered for comparison of HFNC vs. COT due to early termination of the trial and crossover among the treatment groups.

A recent multicenter, retrospective cohort study reported by Marti et al. [37] also explored the effectiveness of different types of noninvasive respiratory support in non-ICU patients with severe COVID-19. The outcomes of this trial showed that NIV was associated with a higher risk of intubation or death compared with HFNC (HR 2.01; 95% CI: 1.32–3.08, *p* = 0.001), while the rates were similar for CPAP and HFNC (HR: 0.97; 95% CI: 0.63–1.50, *p* = 0.891). Several factors may explain the poorer outcomes of NIV, including the high expired tidal volume, which induces superimposed ventilator-induced lung injury [38] and patient–ventilator asynchronies. Moreover, NIV needs highly trained staff.

However, the results of the study by Marti et al. are not in line with those of Franco et al. [39], which showed no differences in the mortality and intubation rates between three types of noninvasive respiratory support (NIV, CPAP, HFNC) in patients with severe COVID-19. On the other hand, an Italian multicenter RCT conducted in four ICUs showed that helmet NIV was associated with a lower endotracheal intubation rate than HFNC (30% vs. 51%; difference, −21% (95% CI, −38% to −3%); *p* = 0.03) [40]. The results of this Italian study indirectly support the report by Patel et al. [41], which showed that helmet NIV was associated with lower endotracheal intubation and 90-day mortality rates than face mask NIV among patients with ARDS. The better outcomes of helmet NIV than face mask NIV may partly be explained by the more effective delivery of higher levels of PEEP.

In conclusion, HFNC may be associated with a lower intubation rate than COT for patients with severe COVID-19; however, there is insufficient evidence to compare the outcomes of HFNC, NIV, and CPAP in patients with severe COVID-19. Moreover, the results of studies in patients receiving NIV from a face mask may not be extrapolated to patients receiving NIV with a helmet. Thus, additional prospective RCTs are required to compare the outcomes of HFNC, CAP, and NIV delivered via different interfaces.

### 4.6. HFNC Combined with the Awake Prone Position

IMV combined with the prone position has been proven to improve both oxygenation and survival in patients with moderate to severe ARDS [42,43,44]. The physiological effects of the prone position include recruiting collapsed lung tissues [45], improving V/Q mismatch [46], and preventing ventilator-induced lung injury [47]. The prone position has also been demonstrated to improve oxygenation in spontaneously breathing non-intubated patients with ARDS [48,49,50]. The prone position combined with various types of noninvasive respiratory support was also widely used in patients with severe COVID-19.

Caputo et al. [51] conducted an observational cohort study in an emergency department (ED), which included 50 COVID-19 patients with hypoxemia. None of the patients were intubated, but all patients were placed in the prone position. They found that early prone positioning had a positive effect on SpO_2_. A similar result was also reported by another cohort study from Italy, in which most patients were treated with helmet CPAP (79%) [52]. The same Italian study also found that half of those responding to the prone position could maintain oxygenation for at least one hour after resupination.

A meta-analysis of observational studies reported that the awake prone position (APP) improved oxygenation in non-intubated COVID-19 patients with hypoxemia, but there was no evidence that APP reduced the rates of intubation or mortality [53]. Thus, it is not clear if the physiological benefit (improved oxygenation) of APP translates into the clinical benefits of reduced intubation or mortality in patients with COVID-19-related AHRF. To address this issue, Ferrando et al. [54] performed a multicenter, adjusted cohort study of 199 patients with COVID-19-related AHRF, all of whom received HFNC, and 55 (27.6%) received HFNC combined with APP. Compared with HFNC alone, HFNC combined with APP was not associated with a lower risk of intubation (RR: 0.87; 95% CI: 0.53–1.43, *p* = 0.60), and 28-day mortality was similar (RR: 1.04; 95% CI: 0.40–2.72, *p* = 0.92). Although the HFNC combined with APP group only included patients placed in the prone position for more than 16 h/day, the authors did not mention whether the patients in the only-HFNC group received APP. Thus, if the patients in the only-HFNC group also received APP (less than 16 h/day), the differences between the two groups may not be large enough to lead to significant differences in the intubation or mortality rates. In addition, the timing of APP added to HFNC was not mentioned. Thus, it is possible that the clinicians in this study used APP as a rescue therapy (i.e., late use) rather than an early intervention.

The first RCT to explore whether APP could reduce the intubation rate was the PROFLO multicenter trial in Sweden, which included 75 patients with COVID-19 hypoxemic respiratory failure, 36 of whom were assigned to the prone group, and 39 to the control group [55]. Not all of the patients received HFNC: only 86% in the prone group and 74% in the control group. The aim was for the patients in the prone group to achieve at least 16 h of APP per day. APP was not prohibited in the control group, but was not encouraged, leaving the clinicians to decide whether to use APP or not. Overall, APP was not associated with a lower intubation rate compared to the control group (33% vs. 33%, HR: 1.01, 95% CI: 0.46–2.21, *p* = 0.99). Only 6% of patients in the prone group achieved the goal of 16 h of APP per day; the daily median duration of the prone position was 9.0 h. However, the daily median duration of the prone position in the control group was 3.4 h. Thus, the difference in the number of hours of APP between the two groups may not be large enough to lead to a significant difference in the intubation rates.

The largest RCT recently published by Ehrmann [56] was a multinational meta-trial that contained 1126 patients with COVID-19-related AHRF, all of whom received HFNC. This meta-trial demonstrated that, compared with standard care, APP could reduce treatment failure (composite outcome: intubation or death) at day 28 (APP vs. standard: 40% vs. 46%, RR 0.86; 95% CI: 0.75–0.98) and was associated with a lower risk of intubation (HR 0.75; 95% CI: 0.62–0.91); however, mortality was similar (HR: 0.87; 95% CI: 0.68–1.11). Moreover, the incidence of adverse events was low and similar in both groups. This meta-trial also found significant improvements in respiratory parameters (SpO_2_/FiO_2_, RR, ROX index) in the first session of the prone position, and these improvements were sustained, even after resupination.

The encouraging result of a reduced risk of intubation in the RCT by Ehrmann [56] is not in line with the findings of the previous PROFLO study. Unlike the PROFLO study, the standard care group in the meta-trial had a median daily prone position duration of 0 h per day, which resulted in a greater difference between the two groups. In a subsequent American trial, one of the members of the meta-trial published a post hoc analysis to explore whether early or late application of APP influences the outcome of patients with COVID-19-related AHRF [57]. The post hoc analysis included 125 patients with COVID-19-related AHRF, all of whom received HFNC. In this analysis, early APP was defined as starting APP within 24 h of initiation of HFNC. The post hoc analysis showed that, compared with late APP, early APP was associated with lower mortality (26% vs. 45%, *p* = 0.039); however, the intubation rate was similar between groups. Thus, this post hoc analysis can be used to form hypotheses for further research.

Recently, an RCT was conducted in Mexico by another of the meta-trial members [58]. The aim of this study was to explore the effect of the duration of APP on the outcome of 430 patients with COVID-19-related AHRF, all of whom were treated with HFNC. Compared with standard care, APP was associated with a lower intubation rate (30% vs. 43%, RR: 0.7; 95% CI: 0.54–0.90, *p* = 0.006), though the mortality rates were similar (APP vs. standard: 33% vs. 37%, RR: 0.89, 95% CI: 0.68–1.15, *p* = 0.37). Post hoc analysis of this Mexican trial showed that more than 8 h of APP per day was associated with a higher chance of treatment success (alive without intubation, adjusted HR: 13.2, 95% CI: 5.4–32.1) and a higher survival rate at 28 days (HR: 5.7, 95% CI: 2.2–14.5). The authors emphasized that APP should be used early and for as long as possible to achieve maximal effects (Table 2).

In summary, although observational studies and the Swedish PROFLO RCT did not suggest that APP reduced the risk of intubation in patients with COVID-19-related AHRF, the more recent multinational meta-trial and Mexican study found that APP combined with HFNC reduces the risk of intubation in addition to improving oxygenation. Although more prospective RCTs are needed to confirm the results of the multinational meta-trial and the Mexican study, it seems reasonable and safe to routinely use APP in patients with COVID-19-related AHRF who have already received HFNC. This conclusion is based on the post hoc analyses, which indicate that early intervention with APP and a longer duration of APP per day result in better outcomes; however, future, well-designed prospective trials are required to confirm these issues. In addition, future studies should investigate whether APP combined with different types of noninvasive respiratory support lead to different outcomes in patients with COVID-19-related AHRF.

### 4.7. Aerosol Dispersion and Aerosol Generation Risk of HFNC

Although HFNC has been widely used in patients with COVID-19 with respiratory failure, HFNC is believed to increase the production of aerosols and thus enhance the spread of COVID-19. However, there is no evidence to support this suggestion, and application of HFNC avoids more dangerous procedures such as intubation, which has been proven to increase the spread of COVID-19. HFNC and other types of noninvasive respiratory support were regarded as aerosol-dispersion procedures rather than aerosol-generating procedures [59].

Hui et al. [60] used the manikin model to simulate the dispersion of exhaled smoke particles during application of HFNC or CPAP. This study showed that the exhaled air dispersion distance increased as the HFNC flow rate increased (65 ± 15 mm for normal lung conditions under 10 L·min^−1^ HFNC, 172 ± 33 mm for normal lung conditions under 60 L·min^−1^ HFNC), and the exhaled air dispersion distance decreased as the severity of lung injury increased (172 ± 33 mm for normal lung conditions under 60 L·min^−1^ HFNC, 48 ± 16 mm for severe lung injury under 60 L·min^−1^ HFNC). Likewise, the exhaled air dispersion distance increased as the pressure of CPAP increased. In addition, the distance of spread was greater for CPAP than HFNC. Li et al. [61] reviewed these three studies [60,62,63] and concluded that the risks of both aerosol generation and dispersion were similar for HFNC and oxygen masks. Moreover, both HFNC and O_2_ masks were associated with a shorter dispersion distance than non-rebreathing masks and venturi masks.

Gaeckle et al. [64] performed a study of ten healthy subjects in a negative-pressure room to evaluate the influence of various types of noninvasive respiratory support on exhaled particles. This study also explored the effects of different maneuvers—including normal breathing, talking, deep breathing, and coughing—on aerosol generation. Their results showed that coughing increased the number of exhaled particles compared to normal breathing, talking, and deep breathing. Moreover, the numbers of exhaled particles were similar for different types of noninvasive respiratory support. The authors concluded that HFNC and other types of noninvasive respiratory support were not associated with increased aerosol generation risk. However, different breathing patterns and coughing increase the risk of aerosol generation.

Jermy et al. [65] further supported the idea that different breathing patterns could influence the risk of aerosol generation. Their study found that snorting, coughing, or sneezing led to the release of 200–1000 times more particles than quiet breathing. However, another study from Singapore of five healthy subjects found that the droplet dispersion distance was higher when subjects were coughing under 60 L·min^−1^ HFNC (mean distance without HFNC: 2.48 m; mean distance with 60 L·min^−1^ HFNC: 2.91 m) [66]. The high flow of air that leaks from the opening of the mouth during coughing may explain the increased dispersion distance under 60 L·min^−1^ HFNC. Thus, it is important to employ measures other than a negative-pressure room to further reduce the distance of aerosol dispersion associated with HFNC. Hamada et al. [67] demonstrated that wearing a surgical mask could prevent particle dispersion when healthy subjects receiving HFNC were coughing. Later, Li et al. [68] found that placing a surgical mask on patients with COVID-19 receiving HFNC could reduce the concentration of aerosol particles in the surroundings.

In summary, the aerosol dispersion risk of HFNC is not greater than COT. Treatment in a negative-pressure room combined with the patients wearing a surgical mask during HFNC could further decrease the aerosol dispersion risk.

## 5. Discussion

HFNC is an open system that allows patients with severe COVID-19 to speak, cough up phlegm, and eat a meal. On the contrary, it is difficult for patients with severe COVID-19 to speak, cough up phlegm, and eat a meal when they are given NIV or IMV. In addition, facial pressure ulcers may develop when an oronasal mask is used for interface of NIV. Patient–ventilator asynchronies may happen when patients are given IMV or NIV, which is not the case with HFNC. Moreover, HFNC is more tolerable and comfortable for patients than NIV and IMV. HFNC may cooperate with other noninvasive respiratory support (e.g., NIV or CPAP) rather than compete with them in treatment of patients with severe COVID-19. Due to aforementioned reasons, HFNC may be suggested as a very good first line therapy in treating patients with severe COVID-19 or be considered a rescue therapy if those patients do not tolerate first-line NIV or CPAP. Bonnesen et al. [69] suggested the use of a step-by-step approach in treating patients with severe COVID-19 in which CPAP was the first-line therapy, and HFNC may be used as a rescue therapy if patients could not tolerate CPAP. There were also reports describing HFNC as a first-line treatment in patients with severe COVID-19 and NIV as a rescue therapy after HFNC failed [18,70]. Whether or not HFNC is a first-line treatment for a step-by-step approach in treating patients with severe COVID-19, it plays an important role because of its great comfort for the patients. However, well-designed prospective trials are needed to compare different stepwise treatment approaches in the future.

## 6. Conclusions

Due to its physiological benefits, HFNC may increase oxygenation, reduce the risk of intubation, and provide satisfactory comfort for patients with COVID-19-related AHRF. HFNC could also alleviate resource constraints and reserve ventilators for the most severely ill patients. However, close monitoring using the ROX index or other respiratory parameters is important to avoid delayed intubation. HFNC combined with early APP may provide better physiological and clinical outcomes in patients with COVID-19. Although HFNC may increase the distance of aerosol dispersion when the flow rate is high or patients are coughing, placing a surgical mask on the patient’s nose and mouth could reduce the risk of transmission to medical staff. Although our manuscript is a review, it fills a gap as we provided precise descriptions of the existing literature, we analyzed and suggested some limitations for each reported study, we proposed explanations for the divergence in the conclusions of the different studies, and we finally suggested future research directions.

## Figures and Tables

**Table 1:** Summary of the difference between early and late intubation for COVID-19 patients.

Authors	Design	N	Definition of Intubation Group	Main Results
Hyman et al. [19]	Retrospective cohort study	755	NA	Intubation increased the in-hospital mortality rate by 1.03-fold per day of delay (adjusted HR, 1.03; 95% CI, 1.01–1.05).
Papoutsi et al. [20]	Meta-Analysis	8944	Early: intubation within 24 h of admission in the ICU. Late: intubation after 24 h from ICU admission.	Mortality: early vs. late: 45.4% vs. 39.1%; RR: 1.07, 95% CI: 0.99–1.15, *p* = 0.08.
Baek et al. [21]	Retrospective cohort study	133	Early: intubation within 48 h of HFNC initiation. Late: intubation after 48 h from HFNC initiation.	Mortality: early vs. late: 38.0% vs. 65.0%, *p* = 0.041.
Candel et al. [22]	Retrospective cohort study	272	Early: intubation within 48 h of HFNC initiation. Late: intubation after 48 h from HFNC initiation.	Mortality: early vs. late: 39.3% vs. 53.2%, *p* = 0.18.

**Table 2:** Summary of the effectiveness of awake prone position (APP) for COVID-19 patients.

Authors	Design	N	HFNC, Percentage	Duration of APP per Day	Main Results
Ferrando et al. [52]	Adjusted cohort study	199	APP group: 100% Control group: 100%	APP group: >16 h Control group: not mentioned	Risk of intubation (Prone group vs. Control group) RR: 0.87; 95% CI: 0.53–1.43, *p* = 0.60. 28-day mortality (Prone group vs. Control group) RR: 1.04; 95% CI: 0.40–2.72, *p* = 0.92.
Rosén et al. [53]	RCT	75	Control group: 74% Prone group: 86%	APP group: median 9.0 h [4.4; 10.6] Control group: median 3.4 h [1.8; 8.4]	Intubation rate: APP vs. Control group: 33% vs. 33%, HR: 1.01, 95% CI: 0.46–2.21, *p* = 0.99.
Ehrmann et al. [54]	RCT (multinational meta-trial)	1126	APP group: 100% Standard care: 100%	APP group: median 5·0 h [1·6; 8·8] Standard care: median 0 h [0;0]	Treatment failure at day 28: APP vs. Standard: 40% vs. 46%, RR 0.86; 95% CI: 0.75–0.98. Risk of intubation: APP vs. Standard: HR 0.75; 95% CI: 0.62–0.91. Mortality: APP vs. Standard: HR: 0.87; 95% CI: 0.68–1.11.
Kaur et al. [55]	Post hoc analysis for an RCT (American trial)	125	Early APP: 100% Late APP: 100%	Early APP: median 5.07 h (2–9.05) Late APP: median 3 h (1.09–5.64)	Mortality: Early vs. Late: 26% vs. 45%, *p* = 0.039. Risk of intubation: Early vs. Late: 37% vs. 42.4%, *p* = 0.58.
Ibarra-Estrada et al. [56]	RCT (Mexican trial)	430	APP group: 100% Standard care: 100%	APP group: median 2·5 h [0·7; 6·9] Standard care: 0 h [0;0]	Risk of intubation: APP vs. Standard: 30% vs. 43%, RR: 0.7; 95% CI: 0.54–0.90, *p* = 0.006. Higher chance of treatment success if APP > 8 h/day: adjusted HR: 13.2, 95% CI: 5.4–32.1. Higher survival rate at 28 days if APP > 8 h/day: HR: 5.7, 95% CI: 2.2–14.5.

## Data Availability

Not applicable.
